# Age at onset determines severity and choice of treatment in early rheumatoid arthritis: a prospective study

**DOI:** 10.1186/ar4540

**Published:** 2014-04-14

**Authors:** Lena Innala, Ewa Berglin, Bozena Möller, Lotta Ljung, Torgny Smedby, Anna Södergren, Staffan Magnusson, Solbritt Rantapää-Dahlqvist, Solveig Wållberg-Jonsson

**Affiliations:** 1 Institution of Public Health and Clinical Medicine/Rheumatology, Umeå University, SE 901 85 Umeå, Sweden; 2 Institution of Public Health and Clinical Medicine/Rheumatology, Sunderbyn Unit, Umeå University, SE 901 85 Umeå, Sweden; 3 Institution of Public Health and Clinical Medicine/Rheumatology, Östersund Unit, Umeå University, SE 901 85 Umeå, Sweden; 4 Institution of Public Health and Clinical Medicine/ Rheumatology, Sundsvall Unit, Umeå University, SE 901 85 Umeå, Sweden; 5 Department of Public Health and Clinical Medicine University Hospital, SE 901 85 Umeå, Sweden

## Abstract

**Introduction:**

Disease activity, severity and comorbidity contribute to increased mortality in patients with rheumatoid arthritis (RA). We evaluated the impact of age at disease onset on prognostic risk factors and treatment in patients with early disease.

**Methods:**

In this study, 950 RA patients were followed regularly from the time of inclusion (<12 months from symptom onset) for disease activity (erythrocyte sedimentation rate (ESR), C-reactive protein (CRP), tender and/or swollen joints, Visual Analogue Scale pain and global scores, and Disease Activity Score in 28 joints (DAS28)) and function (Health Assessment Questionnaire (HAQ)). Disease severity, measured on the basis of radiographs of the hands and feet (erosions based on Larsen score), extraarticular disease, nodules, and comorbidities and treatment (disease-modifying antirheumatic drugs (DMARDs), corticosteroids, biologics and nonsteroidal anti-inflammatory drugs) were recorded at the time of inclusion and at 5 years. Autoantibodies (rheumatoid factor, antinuclear antibodies and antibodies against cyclic citrullinated peptides (ACPAs)) and genetic markers (human leucocyte antibody (HLA) shared epitope and protein tyrosine phosphatase nonreceptor type 22 (PTPN22)) were analysed at the time of inclusion. Data were stratified as young-onset RA (YORA) and late-onset RA (LORA), which were defined as being below or above the median age at the time of onset of RA (58 years).

**Results:**

LORA was associated with lower frequency of ACPA (*P* < 0.05) and carriage of PTPN22-T variant (*P* < 0.01), but with greater disease activity at the time of inclusion measured on the basis of ESR (*P* < 0.001), CRP (*P* < 0.01) and accumulated disease activity (area under the curve for DAS28 score) at 6 months (*P* < 0.01), 12 months (*P* < 0.01) and 24 months (*P* < 0.05), as well as a higher HAQ score (*P* < 0.01) compared with YORA patients. At baseline and 24 months, LORA was more often associated with erosions (*P* < 0.01 for both) and higher Larsen scores (*P* < 0.001 for both). LORA was more often treated with corticosteroids (*P* < 0.01) and less often with methotrexate (*P* < 0.001) and biologics (*P* < 0.001). YORA was more often associated with early DMARD treatment (*P* < 0.001). The results of multiple regression analyses supported our findings regarding the impact of age on chosen treatment.

**Conclusion:**

YORA patients were more frequently ACPA-positive than LORA patients. LORA was more often associated with erosions, higher Larsen scores, higher disease activity and higher HAQ scores at baseline. Nevertheless, YORA was treated earlier with DMARDs, whilst LORA was more often treated with corticosteroids and less often with DMARDs in early-stage disease. These findings could have implications for the development of comorbidities.

## Introduction

Rheumatoid arthritis (RA) is a chronic inflammatory disease with an age-related incidence. It is present in all ethnic populations and at all ages, with prevalence increasing together with age and reaching approximately 2% in a geriatric population [1]. RA is a progressive, destructive joint disease leading to reduced physical function, impaired quality of life and increased risks for comorbidity and premature death if left untreated [2-8]. The presence of different autoantibodies, particularly antibodies against cyclic citrullinated peptides and proteins (ACPAs) indicates an unfavourable prognosis regarding the disease course [9,10]. Age at disease onset has been implicated as an indicator of disease activity, disease severity, comorbidity and effective pharmacological treatment [11-15]. Researchers in previous studies in this field have reported that patients with late-onset RA (LORA) have a more benign form of the disease than those with young-onset RA (YORA) [16-19]. Some of these studies were performed before the 1987 American Rheumatism Association criteria [20] were introduced, and some patients had probably received an alternative diagnosis, *for example*, polymyalgia rheumatica. The results of studies conducted during recent decades that have directly compared LORA and YORA have been inconsistent with earlier studies. Despite differing cutoff ages, the prognosis for LORA patients has not been reported to be very different from that for YORA patients in terms of Disease Activity Score in 28 joints (DAS28), Health Assessment Questionnaire (HAQ) score and the degree of radiographically detected joint damage. Some researchers have even described a poorer prognosis in patients with LORA [11,12,21,22]. Few of these previous publications included individuals with very early RA.

According to published studies, elderly RA patients do not receive the same treatment as younger RA patients, despite having either greater or equivalent disease activity [13,15,23-25]. These data are at variance with YORA patients, who are generally treated adequately and in accordance with national guidelines [26-28]. A rapid diagnosis of genuine RA is important, however, to initiation of early treatment and consequently to achieve the best prognosis and clinical outcome for the patient [29-32].

We hypothesized that age at the onset of RA influences prognosis and any treatment prescribed. Thus, our aim in the present study was to evaluate the impact of age at disease onset on prognostic risk factors, disease progression and pharmacological treatment in a large inception cohort of patients with RA from northern Sweden.

## Methods

To identify potential participants in our study, we referred to the nationwide *Swedish Early RA Registry*, a part of the Swedish Rheumatology Quality Register [33]. All eligible patients from the four northernmost counties of Sweden diagnosed with early RA (*that is*, symptomatic for <12 months) since December 1995 who met the 1987 American Rheumatism Association classification criteria [20] were consecutively included in our large observational study on the progression of RA, including complications and comorbidities. By February 2011, we had registered 950 patients (649 women and 301 men) with newly diagnosed incident early RA who had been included in the study at the time of RA diagnosis (baseline, T0). From among these patients, 665 had been followed for 5 years (T5). All patients had been assessed regularly with a clinical examination performed by their local rheumatologists during this follow-up period, together with routine laboratory tests and radiographs of their hands and feet. The regional Ethics Committee at the University Hospital of Umeå approved this study, and all participants gave their written informed consent in accordance with the Declaration of Helsinki.

According to the criteria for inclusion of individuals in the *Swedish Early RA Registry*, the following data were recorded at baseline and at 6, 12, 18, 24, 36 and 60 months: the 28-joint count of tender joints (TJC) and swollen joints (SJC); a Visual Analogue Scale (VAS) score for pain and the patient’s global assessment; completion of a HAQ [34]; and measurement of inflammatory markers, *that is*, erythrocyte sedimentation rate (ESR) and C-reactive protein (CRP). The DAS28 score was then calculated [35]. These data were downloaded for the purposes of this study.

The therapeutic response was calculated according to the European League Against Rheumatism (EULAR) response criteria using the DAS28 score, thereby defining a response to therapy as “good”, “moderate” or “none” [36]. The presence of autoantibodies, *that is*, rheumatoid factor (RF) and antinuclear antibodies (ANAs), was assessed at baseline according to the routine protocols in current use at each of the participating hospitals. ACPAs were analysed at baseline using enzyme-linked immunoassays for anti–cyclic citrullinated peptide 2 antibodies (Euro Diagnostica, Malmö, Sweden). Genotyping for protein tyrosine phosphatase nonreceptor type 22 (PTPN22) 1858C/T polymorphisms and patients positive for shared-epitope (SE) human leucocyte antigen major histocompatibility complex, class II, DR β1 chain (HLA-DRB1*0401,0404,0405,0408 alleles), was performed as previously described [37-39].

Radiographs of the hands, wrists and feet were obtained at baseline and at 24 months according to the recommendations of the *Swedish Early RA Registry*. However, radiographs are randomly missing from one centre and completely from three of the participating centres. The radiographs were graded by two rheumatologists (SRD and EB) specially trained in the evaluation of radiographs according to the Larsen score [40]. Radiological progression was defined as being present if the Larsen score at 24 months minus the Larsen score at baseline was ≥3 (dichotomized at the median value of this difference). The presence of erosions at T0 and at 24 months was also registered.

Additionally, all patient records were carefully examined and data collected according to a study protocol regarding co-morbidity and treatment, both at inclusion (T0) and after five years (T5). The patients also completed a self-reported questionnaire on comorbidity at T0 and T5 to increase further the validity of the collected data. Recorded variables were cardiovascular disease (CVD) as previously described in detail [41], the presence of hypertension (HT) and/or diabetes mellitus (DM), smoking (currently and previously), the presence of rheumatoid nodules and extra-articular disease [42]. Cumulated pharmacological treatment was registered (months before inclusion and during the follow-up period) regarding corticosteroids and disease modifying anti-rheumatic drugs (DMARDs, *that is*, methotrexate, sulphasalazine, chloroquine, azathioprine, mycophenolate mofetil, myocrisin, auranofin, cyclosporine, leflunomide, alkylating cytotoxic agents) including biological agents (etanercept, adalimumab, infliximab, anakinra, rituximab). Treatment with non-steroidal anti-inflammatory drugs (NSAIDs), before inclusion (T0) and any period during the follow up period (T5), was recorded simply as “yes” or “no”.

### Statistical analysis

Descriptive data collected at baseline and at 5 years are presented as the mean (± standard deviation (SD)) or as a percentage. Data analyses were based on stratification of the patients according to median age (58 years) at the time of disease onset as YORA (*that is*, <58 years) and LORA (≥58 years). In patients with a single data point missing from the *Swedish Early RA Registry* data, *that is*, ESR, CRP, TJC/SJC, VAS score or HAQ score, the previous value was used to impute data once for each inflammatory variable assessed up to 24 months. The proportions of imputations for each variable and registration were 3.6% from 0 to 6 months; however, that figure increased somewhat up to 24 months and is estimated to be, at most, 16%. Disease activity, based on DAS28 score, was calculated according to the trapezoid model [43] to evaluate the total burden of disease activity over time, referred to as the area under the curve (AUC) of DAS28 scores, at 6, 12 and 24 months after inclusion into the study. Comparisons of data between YORA and LORA were made with independent and dependent *t*-tests and χ^2^ tests as appropriate. Multiple logistic regression analyses were first used to evaluate the impact of known prognostic factors at baseline, *that is*, disease activity (ESR), ACPA, comorbidity and sex, together with age at disease onset, on a chosen treatment, *that is,* DMARD treatment within 3 months from disease onset and corticosteroid treatment, as dependent variables. Next, we tested the impact of a chosen treatment, adjusted for prognostic risk factors, on disease outcome, *that is*, radiological progression as measured by the difference in the Larsen score (Δ = 0 to 24 months) dichotomized for the median value as the dependent variable. Variables were initially chosen for multiple modelling based on clinical assumptions and only secondarily using the results from simple statistical analyses (*P* < 0.05). Correlation tests and standard errors indicated no collinearity for the included variables in any of the models. All *P*-values are two-sided, and *P*-values ≤0.05 were considered to be statistically significant. All calculations were performed using the IBM SPSS Statistics 21.0 software program (SPSS, Chicago, IL, USA).

## Results

A total of 950 patients diagnosed with early RA were included between December 1995 and February 2011 at the five participating rheumatology centres. By 28 February 2011, 665 patients had been followed for 5 years (T5). All patients could be tracked to the 5-year follow-up time point. The median age at disease onset in this cohort of patients was 58 years (range = 18 to 89 years).

### Demographic data at baseline and after 5 years

Descriptive data for all patients, which were divided into the two groups, *that is*, YORA (<58 years) and LORA (≥58 years) are presented in Table 1. The total study group of 950 patients at baseline (T0) comprised 357 women (75.2%) and 118 men (24.8%) in the YORA patient group and 292 women (61.5%) and 183 men (38.5%) in the LORA group. Among the patients who reached the 5-year follow-up point (T5) (*that is*, *n* = 665), there were 262 women and 89 men in the YORA group and 197 women and 117 men in the LORA group. The mean duration (±SD) from the first sign of symptoms of rheumatoid disease until inclusion into the *Swedish Early RA Registry* was 6.9 (±3.5) months for YORA patients and 6.5 (±3.2) months for LORA patients (*P* = 0.048) with no sex differences, *that is*, 6.5 (±3.3) months for men and 6.8 (±3.6) months for women (*P* = 0.265).

**Table 1:** **Descriptive data for 950 patients with early rheumatoid arthritis at time of inclusion and at 5-year follow-up**^**a**^

**Variables**	**YORA (*N *** **= 475)**	**LORA (*N *** **= 75)**	***P*-value**
Age at onset, yr	<58	≥58	
Female, *n* (%)	*357/475* (75.2)	*292/475* (61.5)	
Disease duration (mo)	6.90 (3.42)	6.45 (3.20)	<0.05
RF+, *n* (%)	*354/466* (75.9)	*349/468* (75.6)	NS
ANA+, *n* (%)	*91/377* (24.2)	*69/352* (19.6)	NS
ACPA+ *n* (%)	*313/429* (72.9)	*267/411* (64.9)	<0.05
PTPN22 T variant, *n* (%)	*155/405* (38.3)	*106/381* (27.8)	<0.01
HLA-SE, shared epitope, *n* (%)	*237/405* (58.5)	*219/381* (57.5)	NS
ESR at T0, mm/h (*n* = *948)*	26.0 (21.5)	34.3 (24.2)	<0,001
CRP at T0 mg/L (*n* = *888)*	18.3 (23.7)	23.0 (24.6)	<0.01
DAS28 at T0, (*n* = 788)	4.5 (1.5)	4.8 (1.4)	<0.01
AUC DAS28 (6 mo)^b^	24.5 (7.2)	25.9 (7.0)	<0.01
AUC DAS28 (12 mo)^b^	44.8 (13.4)	47.7 (13.2)	<0.01
AUC DAS28 (24 mo)^b^	84.2 (26.8)	89.0 (23.3)	<0.05
HAQ at T0, (*n* = 796)	0.81 (0.6)	0.92 (0.6)	<0.01
Tender joints at T0 (*n* = 811)	6.3 (5.6)	6.5 (5.7)	NS
Swollen joints at T0, (*n* = 812)	6.8 (5.2)	7.3 (5.2)	NS
VAS pain at T0 (mm), (*n* = 800)	43.5 (25.5)	43.4 (25.9)	NS
VAS global at T0 (mm) (*n* = 800)	44.0 (25.8)	45.0 (25.5)	NS
Smoker (ever) T0, *n* (%)	*287/443* (64.8)	*300/447* (67.1)	NS
Ex-RA ≤ T5, *n* (%)^c,d^	*13/344* (3.8)	*14/313* (4.5)	NS
Nodules ≤ T5, *n* (%)^c^	*60/312* (19.2)	*38/275* (13.8)	0.079
DMARDs within 3 mo after T0, *n* (%)^c,e^	*325/346* (93.9)	*267/311* (85.9)	<0.001
DMARDs (ever) ≤T5, *n* (%)^c^	*344/348* (98.9)	*299/310* (96.5)	<0.05
Methotrexate (ever) ≤T5, *n* (%)^c^	*313/346* (90.5)	*253/311* (81.4)	<0.001
Biologics (ever) ≤T5, *n* (%)^c^	*84/342* (24.6)	*23/310* (7.4)	<0.001
NSAID T0, *n* (%)	*317/455* (69.7)	*297/466* (63.7)	0.056
NSAID ever ≤T5, *n* (%)^c^	*307/340* (90.3)	*233/307* (75.9)	<0.001
COX-2-inhibitors at T0, *n* (%)	*48/459* (10.5)	*64/468* (13.7)	NS
COX-2-inhibitors (ever) ≤T5, *n* (%)^c^	*95/342* (27.8)	*83/309* (26.8)	NS
Corticosteroids T0 to T5 (mo)^c^	20.3 (23.8)	24.7 (23.8)	<0.01
Corticosteroids (ever) ≤T5 (%)^c^	*230/346* (66.5)	*233/309* (75.4)	<0.01
Hypertension at T0, *n* (%)	*56/471* (11.9)	*199/474* (41.9)	<0.001
DM at T0, *n* (%)	*22/464* (4.7)	*54/470* (11.5)	<0.001
CVD before T0, *n* (%)^f^	*17/471* (3.6)	*93/474* (19.6)	<0.001
CVD related comorbidity at T0, *n* (%)^g^	*86/475* (18.1)	*238/475* (50.1)	<0.001
Larsen score 0 (mo) (*n* = 437)	5.3 (5.8)	8.5 (6.2)	<0.001
Larsen score 24 (mo) (*n* = 381)	9.5 (9.1)	12.5 (8.2)	<0.001
Erosions 0 mo, *n* (%)	*85/212* (40.1)	*105/192* (54.7)	<0.01
Erosions 24 mo, *n* (%)	*107/173* (61.8)	*119/158* (75.3)	<0.01
Δ Larsen score 0 to 24 mo ≥3, *n* (%)	*103/207 (*49.8)	*97/173 (*56.1)	0.131

^a^ACPA, Anticyclic citrullinated peptide/protein; ANA, Antinuclear antibody; AUC, Area under the curve; CVD, Cardiovascular disease; CRP, C-reactive protein; DAS28, Disease Activity Score in 28 joints; DM, Diabetes mellitus; DMARD, Disease-modifying antirheumatic drug**;** ESR, Erythrocyte sedimentation rate; HAQ, Health Assessment Questionnaire; LORA, Late-onset rheumatoid arthritis; NS, Not significant; NSAID, Nonsteroidal anti-inflammatory drug; PTPN22, Protein tyrosine phosphatase nonreceptor type 22; RA, Rheumatoid arthritis; RF, Rheumatoid factor; SD: Standard deviation; SE, Shared epitope; VAS, Visual Analogue Scale; YORA, Young-onset rheumatoid arthritis. ^b^AUC for DAS28 at 6 months after inclusion, YORA/LORA (*n* = 367/343); AUC for DAS28 at 12 months after inclusion, YORA/LORA (*n* = 308/281); AUC for DAS28 at 24 months after inclusion, YORA/LORA (*n* = 231/199). ^c^Patients followed for 5 years (*n* = 665). ^d^Criteria used for severe extraarticular manifestations: pericarditis, pleuritis, interstitial lung disease, Felty’s syndrome, neuropathy, scleritis/episcleritis, glomerulonephritis, major cutaneous vasculitis and vasculitis involving other organs [42]. ^e^DMARD treatment started within 3 months from baseline (T0). ^f^CVD comorbidities as previously described in detail [41]. ^g^CVD-related comorbidity at time of inclusion (T0): CVD, hypertension or DM present before T0.

### Autoantibodies, genetic markers and measures of disease activity

Comparison between LORA and YORA patients revealed that LORA was significantly associated with a lower frequency of ACPA (Table 1) and less frequent carriage of the PTPN22 T-variant. ACPA was more common (66%) in the youngest quartile (18 to 47 years of age), with a gradual decrease (65% in patients 48 to 58 years of age and 62% in patients 59 to 66 years of age) to 50% in the oldest quartile (age range = 67 to 89 years). In the present study, the presence of RF and HLA-SE (defined as HLA-DRB1*0401,0404,0405,0408 alleles) was not associated with age at disease onset.

Disease activity, measured by a combination of ESR, CRP and accumulated Disease Activity Score (AUC for DAS28) was higher in LORA patients than in patients with disease onset at a younger age, as was greater reduction in physical function measured by HAQ score at baseline (Table 1). The presence of extraarticular manifestations was similar in both groups, whilst rheumatic nodules tended to be more common in YORA patients.

### Radiographic findings at inclusion and at 2 years

Radiographs of the hands, wrists and feet were taken at baseline for 230 YORA and 207 LORA patients and at 2 years for 208 YORA and 173 LORA patients. LORA was significantly more often associated with the presence of erosions and a higher Larsen score at both time 0 and at 24 months (Table 1). Both YORA and LORA patients exhibited radiological progression as measured by an increase in Larsen scores over time (YORA = 5.31 (±5.8) vs. 9.46 (±9.04) and LORA = 8.14 (±5.81) vs. 12.45 (±8.22); *P* < 0.001 for both). Additionally, the presence of erosions increased significantly between 0 and 24 months in both groups (YORA 40.1% vs. 61.8% and LORA 54.7% vs. 75.3%; *P* < 0.001). However, there was no statistically significant difference between YORA and LORA patients in terms of progression of the Larsen scores or the presence of erosions (data not shown).

### Pharmacological treatment and cardiovascular disease comorbidity at baseline and at 5 years

LORA was associated with less frequent treatment with DMARDs, particularly early in the disease process (Table 1). LORA was also associated with significantly less treatment with methotrexate and biologics and more frequent treatment with corticosteroids (Table 1). A reduction in the DAS28 score during the first 24 months was numerically larger in LORA than in YORA (1.72 (±1.57) vs. 1.44 (±1.71); *P* = 0.058). With regard to EULAR response criteria, 26% of patients with LORA were classified as “nonresponders,” compared with 36.7% of YORA patients, at 12 months (*P* < 0.01). Comorbidity, such as HT, DM and CVD, was present significantly more often in LORA patients (*P* < 0.001) (Table 1).

### Multiple logistic regression models

The impact of age at disease onset was tested in relation to the choice of treatment, *that is*, early treatment with DMARDs (within 3 months following the onset of disease) and with corticosteroids, respectively, in multiple regression models. In baseline models adjusted for sex, ACPA status and disease activity (measured by ESR), YORA was significantly associated with early DMARD treatment (Table 2) and LORA was associated with corticosteroid treatment (Table 3). Age was also related to the chosen drug treatment when tested as a continuous variable (odds ratio (OR) = 0.969, 95% confidence interval (CI) = 0.945 to 0.993, *P* < 0.01 for early DMARD treatment; OR = 1.021, 95% CI = 1.006 to 1.035, *P* < 0.01 for corticosteroid treatment). In addition, the results were similar regarding age at the onset of disease when the data were corrected for baseline comorbidity (OR = 0.371, 95% CI = 0.194 to 0.709, *P* < 0.01 for early DMARD treatment as the dependent variable; OR = 1.453, 95% CI = 0.967 to 2.183, *P* = 0.072 for corticosteroids) (Additional file 1: Table S1 and Additional file 2: Table S2).

**Table 2:** **Age in relation to disease-modifying antirheumatic drug treatment within 3 months after disease onset**^**a**^

**Covariates**	**OR**	**95% CI**	***P*-value**
Age at disease onset^b^	0.403	0.217 to 0.749	<0.01
Female sex	1.002	0.535 to 1.878	0.994
ACPA-positive	1.484	0.812 to 2.712	0.199
ESR (T0), mm/h	1.027	1.009 to 1.045	<0.01

^a^ACPA, Anticyclic citrullinated peptide/protein; CI: Confidence interval; DMARD, Disease-modifying antirheumatic drug; ESR, Erythrocyte sedimentation rate; OR: Odds ratio. ^b^Age at disease onset classified as young-onset rheumatoid arthritis <58 years old and late-onset rheumatoid arthritis ≥58 years old. Data are derived from multiple logistic regression (*n* = 562).

**Table 3:** **Age in relation to corticosteroid treatment**^**a**^

**Covariates**	**OR**	**95% CI**	***P*-value**
Age at disease onset^b^	1.540	1.045 to 2.267	<0.05
Female sex	0.803	0.529 to 1.219	0.304
ACPA-positive	1.127	0.752 to 1.693	0.565
ESR (T0), mm/h	1.011	1.002 to 1.020	<0.05

^a^ACPA, Anticyclic citrullinated peptide/protein; CI: Confidence interval; ESR, Erythrocyte sedimentation rate; OR: Odds ratio. ^b^Age at disease onset classified as young-onset rheumatoid arthritis <58 years old and late-onset rheumatoid arthritis ≥58 years old. Data are derived from multiple logistic regression (*n* = 574).

Regarding radiological progression over 24 months as the response variable, ACPA status and older age at disease onset (LORA) were significantly associated with progression of Larsen score of ≥3 (0 to 24 months) in multiple models adjusted for age, sex, ACPA status, disease activity (based on ESR), comorbidity at baseline and chosen therapy, *that is*, early treatment with DMARDs (Table 4) and corticosteroids (Table 5), respectively. In these multiple models, male sex was significantly related to greater radiological progression at 24 months (Tables 4 and 5).

**Table 4:** **Age in relation to radiological progression**^**a**^

**Covariates**	**OR**	**95% CI**	***P*-value**
Age at disease onset^b^	2.019	1.084 to 3.761	<0.05
Female sex	0.368	0.188 to 0.722	<0.01
ACPA-positive	2.179	1.117 to 4.251	<0.05
ESR (T0), mm/h	1.008	0.995 to 1.022	0.231
CVD-related comorbidity^c^	0.838	0.420 to 1.675	0.671
DMARD treatment within 3 month after T0, yes	1.400	0.594 to 3.298	0.442

^a^ACPA, Anticyclic citrullinated peptide/protein; CI: Confidence interval; CVD, Cardiovascular disease; DMARD, Disease-modifying antirheumatic drug; ESR, Erythrocyte sedimentation rate; OR: Odds ratio. ^b^Age at disease onset stratified as young-onset rheumatoid arthritis <58 years old and late-onset rheumatoid arthritis ≥58 years old. ^c^CVD-related comorbidity at time of inclusion (T0): CVD, hypertension or diabetes mellitus present before T0. *Radiological progression* is defined as the difference between Larsen score at 24 months and Larsen score at baseline (Δ Larsen score) ≥3. Data are derived from multiple logistic regression (*n* = 227).

**Table 5:** **Age in relation to radiological progression**^**a**^

**Covariates**	**OR**	**95% CI**	***P*-value**
Age at disease onset^b^	1.842	1.014 to 3.348	<0.05
Female sex	0.361	0.189 to 0.692	<0.01
ACPA-positive	2.473	1.282 to 4.769	<0.01
ESR (T0), mm/h	1.010	0.997 to 1.024	0.136
CVD-related comorbidity^c^	0.889	0.460 to 1.718	0.727
Corticosteroids (ever) ≤T5, yes	0.892	0.488 to 1.631	0.711

^a^ACPA, Anticyclic citrullinated peptide/protein; CI: Confidence interval; CVD, Cardiovascular disease; DMARD, Disease-modifying antirheumatic drug; ESR, Erythrocyte sedimentation rate; OR: Odds ratio. ^b^Age at disease onset stratified as young-onset rheumatoid arthritis <58 years old and late-onset rheumatoid arthritis ≥58 years old. ^b^CVD-related comorbidity at time of inclusion (T0): CVD, hypertension or diabetes mellitus present before T0. ^c^*Radiological progression* is defined as the difference between Larsen score at 24 months and Larsen score at baseline (Δ Larsen score) ≥3. Data are derived from multiple logistic regression (*n* = 236).

In these multiple models, male sex was significantly related to a greater radiological progression at 24 months (Tables 4 and 5).

## Discussion

Our objective in the present study was to describe the impact of age at the onset of RA on prognostic risk factors, disease severity and chosen treatment early in the disease process. LORA (*that is*, age ≥58 years) was associated with greater disease activity and radiographic changes at disease onset. Also, inflammatory activity over time and functional impairment were greater in patients with LORA. Nevertheless, YORA (*that is*, <58 years of age) was more frequently associated with early treatment with DMARDs and biologics compared with LORA patients, who were treated more often with corticosteroids but less often with DMARDs during the first 3 months following disease onset. These findings, taken together, indicate unequal treatment, which may be important for the prognosis and development of comorbidities in the long term.

In this study, the YORA patients were significantly more often positive for ACPA, an autoantibody that is associated with worse disease progression [9,10]. Furthermore, the prevalence of ACPA decreased with increasing age at onset of RA. The YORA patients were also more frequently carriers of the PTPN22 T-variant compared with LORA patients. In a previous study, we found carriage of the PTPN22 T-variant and presence of ACPA to be independently associated with disease onset at an earlier age (*P* < 0.05 and *P* < 0.01, respectively), whilst the combination of both was associated with an even younger age at disease onset [39]. In the present study, the frequency of RF and HLA-SE positivity was similar for both the YORA and LORA cohorts, which is consistent with previously published findings by van der Heijde and colleagues [22]. We found that LORA was more often associated with the presence of erosions and a higher Larsen score, both at baseline and after 2 years. These findings are also in line with those presented by van der Heijde *et al.*, who found a tendency towards more radiographically detected damage in older patients compared with patients younger than 60 years of age at follow-up [22]. Furthermore, Bukhari *et al.* showed that an older age at the onset of symptoms was associated with an increased occurrence of erosions during the first 12 months in patients diagnosed with early polyarthritis [14]. We also found that a raised HAQ score at baseline was associated with LORA. This finding is similar to that reported by Pease *et al*., who found that patients over 65 years of age had a higher HAQ score at baseline [11], and with results derived from the Norfolk Arthritis Register showing that more advanced age at symptom onset was associated with a “steep trajectory” progression of disability [44]. Taken together, our findings indicate a phenotype more predisposed to progressive disease related to ACPA positivity and PTPN22 polymorphism in patients whose onset of disease occurs at a younger age. Nevertheless, the radiological findings and the functional capacity are worse in patients with a later onset. We also found a worse radiologically detectable disease progression in males, thus confirming our findings in a previous study [45] and meriting further investigation.

A late onset of RA was associated with a higher DAS28 score calculated at baseline. However, a reduction in DAS28 score after 24 months was somewhat greater in patients with LORA in comparison to those with YORA. Also, when the data were stratified for response to therapy using the EULAR response criteria [36], LORA patients had a significantly better response after 12 months than patients with YORA. The LORA patients in the present study were more often treated with corticosteroids, which are known to have a capacity to rapidly reduce disease activity, early in the course of disease progression. Despite receiving such therapy, LORA patients more often had erosions and a higher Larsen score at 24 months. Late onset-disease was associated with less frequent treatment with methotrexate and biologics compared with YORA, as well as with less frequent treatment with DMARDs early in the disease process, *that is*, within 3 months from inclusion into the study. Apparently, treatment with corticosteroids reduced inflammatory activity efficiently but could not compensate for the less frequent DMARD treatment early in the disease course in the patients with late-onset disease in terms of radiological status at 24 months. Radiological progression appeared without regard to the chosen drug treatment. It is conceivable that a more evident impact of the chosen treatment would be demonstrable in a larger cohort or with a longer follow-up period. There was no delay in the diagnosis of the LORA patients, with the disease duration being somewhat shorter in this group. Coexisting osteoarthritis could contribute to the worse radiographic status in LORA patients, as could the possibility of cartilage that is more susceptible to damage in elderly individuals, as has been suggested by others [11,14]. However, our finding of a greater Larsen score cannot be explained simply by the progression of cartilage damage as part of osteoarthritis, because there was also a significant progression of the erosions over time in LORA patients. Thus, the treatment of LORA patients with corticosteroids did not reduce radiological progression, despite the positive effects of these drugs on inflammatory activity. Treatment with low doses of prednisolone as a monotherapy, or in combination with DMARDs, has previously been reported to have positive effects with respect to the remission rate and inhibition of radiographic progression [46-48]. However, contrasting results were reported by Paulus *et al*., who found that monotherapy with low-dose corticosteroids did not prevent radiological progression [49].

Earlier studies generally concluded LORA to be a mild disease with a good prognosis [16-19] but several more recent studies have shown equal, or worse, disease activity and severity in older patients compared with younger patients [11-15,22,50]. In, agreement with Swedish guidelines [26], and similar to EULAR [27] and American College of Rheumatology guidelines [28], we treat patients with DMARDs, primarily methotrexate, in combination with low doses corticosteroids if their disease activity is considered to be “high to moderate” at disease onset. Nevertheless, our study shows that age at disease onset influences the choice of pharmacological treatment. According to our results, rheumatologists are less likely to treat LORA patients with DMARDs within 3 months from disease onset compared with YORA. Anderson *et al*. [51] reported that patients with longer disease durations did not respond to treatment to the same extent as patients with early disease, thus giving additional support to the observation that early DMARD therapy modulates inflammation more effectively. One possible explanation for later initiation of DMARD treatment is that LORA patients tend to have more comorbidities and coexisting diseases requiring treatment with other drugs. However, when comorbidity at baseline was also taken into account, the choice of treatment was associated with age at onset of disease.

Early in the disease course, it is easy and efficient to control disease activity by using corticosteroid treatment. Both the clinician and the patient may be pleased with the rapid relief of symptoms achieved with corticosteroid treatment, and there is more concern about the potential side effects of DMARDs in older patients. At times, this clinical decision may be correct, and a risk vs. benefit profile must always be taken into consideration. Treatment with corticosteroids can be precarious in elderly patients and should be evaluated with respect to increased risk of adverse effects, such as osteoporosis, infection, diabetes, peptic ulcers*,* cataract and HT [52]*.* In the current literature, data are still lacking regarding the net effect of corticosteroids in patients with inflammation. Several studies have shown that treatment with methotrexate, sulphasalazine and antimalarial therapy, and other DMARDs is well-tolerated by the elderly [50]. Tutuncu and co-workers showed that toxicity due to methotrexate and biological agents was very low in all patients studied, regardless of age [13]. In that study, patients with LORA were also treated significantly less often with biological agents compared with YORA patients. This observation is consistent with recently published findings of Huscher *et al*. [53]. Recent studies have shown that biological therapy can be used in the treatment of elderly patients, but risk factors for adverse effects should be evaluated extra carefully before starting any therapy [54,55].

The strengths of the present study are the patient group, which consists of a large regional cohort, and its prospective design. The study involves few physicians at each rheumatology centre in northern Sweden. In Sweden, essentially all patients with newly diagnosed RA are referred to a specialist. Thus, the results derived from the present cohort can be regarded as applicable to all patients with early RA. Furthermore, repeated measurement of the parameters associated with inflammation made it possible to take into account variability in disease activity. Conversely, a limitation of the study is its observational nature, with a risk of confounding by indication when evaluating the efficiency of pharmacological treatment. We attempted to adjust for that possibility by using multiple regression modelling in the statistical analyses when evaluating potential covariates in relation to LORA.

## Conclusions

LORA was associated with greater incipient disease activity, reduced function at baseline and more radiological damage at disease onset and over time. Nevertheless, such patients were more often treated with corticosteroids and less often with DMARDs early in the disease process compared with the YORA patients. We propose that patients with LORA should receive the same treatment as younger patients at the onset of disease; however, such patients may require tighter controls to rapidly detect any potential complicating comorbidity or other undesirable side effects of the treatment.

## Abbreviations

ACPA: Anticyclic citrullinated peptide/protein antibody; ANA: Antinuclear antibody; AUC: Area under the curve; CI: Confidence interval; CVD: Cardiovascular disease; COX-2: Cyclooxygenase 2; CRP: C-reactive protein; DAS28: Disease Activity Score in 28 joints; DM: Diabetes mellitus; DMARD: Disease-modifying antirheumatic drug; ESR: Erythrocyte sedimentation rate; EULAR: European League Against Rheumatism; HAQ: Health Assessment Questionnaire; HLA: Human leucocyte antigen; HT: Hypertension; LORA: Late-onset rheumatoid arthritis; NSAID: Nonsteroidal anti-inflammatory drug; OR: Odds ratio; PTPN22: Protein tyrosine phosphatase nonreceptor type 22; RA: Rheumatoid arthritis; RF: Rheumatoid factor; SD: Standard deviation; SE: Shared epitope; SJC: Swollen joint count; TJC: Tender joint count; T0: Baseline (time 0); T5: Patients followed for 5 years; VAS: Visual Analogue Scale.

## Competing interests

The authors declare that they have no competing interests.

## Authors’ contributions

LI participated in the design of the study, collected and registered patient data contributed to the statistical analysis and drafted the manuscript. AS, LL, BM, SM and TS participated in the collection and registration of the patient data and drafted the manuscript. EB participated in the collection and registration of the patient data, evaluated the X-rays and drafted the manuscript. SRD participated in the design of the study, collected patient data, evaluated the X-rays and drafted and revised the manuscript critically. SWJ was the Principal Investigator of the study, designed the investigation and participated in data collection, statistical analysis and drafting of the manuscript. All authors contributed to discussions and read and approved the final manuscript.

## Supplementary Material

Additional file 1: Table S1 Age in relation to disease-modifying antirheumatic drug therapy initiated within 3 months after baseline. Data were assessed by multiple logistic regression analysis (*N* = 562). Click here for file

Additional file 2: Table S2 Age in relation to corticosteroid treatment. Multiple logistic regression data (*N* = 574). Click here for file
