# Supplementary material for: Age at onset determines severity and choice of treatment in early rheumatoid arthritis: a prospective study
Source: Arthritis Res Ther. 2014 Apr 14;16(2):R94. doi: 10.1186/ar4540 (PMC4060263; doi:10.1186/ar4540)
Supplement: Additional file 1: Table S1 — Age in relation to disease-modifying antirheumatic drug therapy initiated within 3 months after baseline. Data were assessed by multiple logistic regression analysis (N = 562). [file ar4540-S1.doc]

| **Table S1. Age in relation to DMARDs within 3 months after T0. Multiple logistic regression. N=562** | | | |
| --- | --- | --- | --- |
| **Co-variates** | **OR** | **CI 95 %** | **p-value** |
| Age at disease onset^1^ | 0.371 | 0.194, 0.709 | <0.01 |
| Sex/male | 1.029 | 0.548, 1.933 | 0.930 |
| ACPA / positive | 1.495 | 0.817, 2.736 | 0.192 |
| ESR (T0)/ mm/h | 1.027 | 1.009, 1.045 | <0.01 |
| CVD related co-morbidity^2^ | 1.328 | 0.680,2.592 | 0.406 |

^1^Age at disease onset stratified as YORA <58 years and LORA ≥58 years;
^2^CVD related co-morbidity at inclusion (T0) = CVD, hypertension or diabetes mellitus present before T0; ACPA, anti-cyclic citrullinated peptide/protein; ESR, erythrocyte sedimentation rate; CVD, cardiovascular disease; OR: odds ratio; CI: confidence interval.
