# Supplementary material for: Age at onset determines severity and choice of treatment in early rheumatoid arthritis: a prospective study
Source: Arthritis Res Ther. 2014 Apr 14;16(2):R94. doi: 10.1186/ar4540 (PMC4060263; doi:10.1186/ar4540)
Supplement: Additional file 2: Table S2 — Age in relation to corticosteroid treatment. Multiple logistic regression data (N = 574). [file ar4540-S2.doc]

| **Table S2. Age in relation to corticosteroid treatment. Multiple logistic regression. (N=574)** | | | |
| --- | --- | --- | --- |
| **Co-variates** | **OR** | **CI 95 %** | **p-value** |
| Age at disease onset^1^ | 1.453 | 0.967, 2.183 | 0.072 |
| Sex/male | 0.812 | 0.535, 1.234 | 0.330 |
| ACPA / positive | 1.136 | 0.755, 1.707 | 0.541 |
| ESR (T0)/ mm/h | 1.011 | 1.002, 1.020 | <0.05 |
| CVD related co-morbidity^2^ | 1.222 | 0.708,1.916 | 0.382 |

^1^Age at disease onset stratified as YORA <58 years and LORA ≥58 years;
^2^CVD related co-morbidity at inclusion (T0)= CVD, hypertension or diabetes mellitus present before T0;
ACPA, anti-cyclic citrullinated peptide/protein; ESR, erythrocyte sedimentation rate; CVD, cardiovascular disease; OR: odds ratio; CI: confidence interval.
